# Petrographical and petrophysical rock typing for flow unit identification and permeability prediction in lower cretaceous reservoir AEB_IIIG, Western Desert, Egypt

**DOI:** 10.1038/s41598-024-56178-z

**Published:** 2024-03-07

**Authors:** Abdelraheim Abo Bakr, Hassan H. El Kadi, Taher Mostafa

**Affiliations:** Geology Department, Faculty of Science, Al-Azhar University, P.O. Box 11884, Nasr City, Cairo, Egypt

**Keywords:** Core data, Petrophysics, Petrography, Rock typing, Meleiha West Deep, Western Desert, Solid Earth sciences, Geology, Geophysics

## Abstract

The primary objective of this study is to identify and analyze the petrophysical properties of the newly investigated AEB_IIIG member reservoir in Meleiha West Deep (MWD) Field and to classify it into different rock types. Additionally, this research intends to develop mathematical equations that may be utilized to estimate permeability in uncored sections of the same well or in other wells where core samples are unavailable. The analysis focused on the pore hole records of ten wells that were drilled in MWD Field. The reservoir levels were identified, and their petrophysical parameters were evaluated using well logs and core data. We were able to recognize seven different types of rocks (petrophysical static rock type 1 (PSRT1) to PSRT7) using petrography data, the reservoir quality index (RQI), the flow zone index (FZI), R35, hydraulic flow units (HFUs), and stratigraphy modified Lorenz (SML) plots. The analysis of the petrophysical data shows that AEB_IIIG has unsteady net pay thicknesses over the area. It has a range of 8–25% shale volume, 12–17% effective porosity, and 72–92% hydrocarbon saturation. The RQI results show that psrt1, psrt2 and psrt3 have a good reservoir quality as indicated by high R35 and helium porosity, respectively. They contribute with more than 75% of the reservoir production. The equation derived for each rock type of AEB_IIIG reservoir can be employed to forecast the permeability value distribution inside the reservoir.

## Introduction

Characterization of rock typing entails the division of a reservoir into distinct zones that possess similar petrophysical and flow features^1–4^. The application of rock typing is prevalent across many industry sectors, encompassing activities such as identifying thief zones during drilling operations, effectively managing zones exhibiting high productivity indices during the production phase, recognizing zones of interest, and constructing resilient numerical reservoir models^5–7^. Rock typing plays a crucial role in the prediction of reservoir parameters, particularly permeability in uncored intervals^8^. The process of coring from multiple wells is frequently necessary and crucial in acquiring fundamental data about the area. Nevertheless, the process of extracting core samples from every well throughout extensive oil fields or from each zone of interest inside a single well is a significant economic challenge. Hence, the utilization of rock typing can serve as a viable approach to mitigate these exorbitant expenditures^9^.

**Figure 1:**
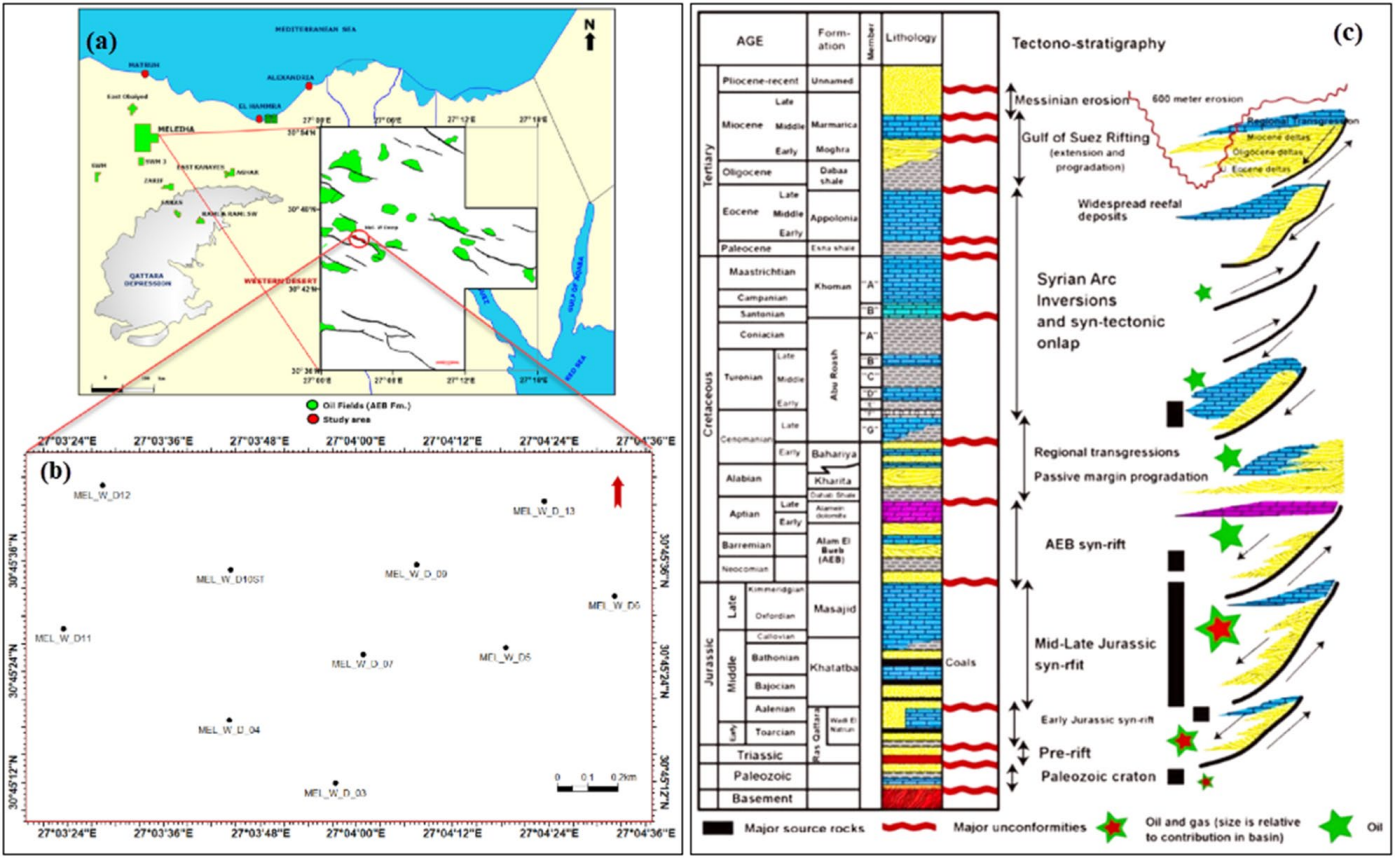
(**a**) Location map of the study area, (**b**) base map indicating the locations of the investigated wells, and (**c**) Lithostratigraphic column of the study area illustrating the tectonostratigraphic evolution of the Shushan basin-fill sedimentary succession^23^.

Western Desert encompasses roughly two-thirds of Egypt's landmass and is located west of the Nile River and Delta. The study area is distinct within Agiba’s Meleiha development lease, which encompasses approximately 700 km^2^ (Fig. 1a). The concession territory is situated within the northern province of the Western Desert, roughly 65 km south of Matrouh city between latitudes 30° 45′ 00″ and 30° 45′ 40″ North and longitudes 27° 03′ 00″ and 27° 05′ 00″ East. It is bordered by Dorra Field in the north-eastern direction, Emry Field in the south direction, and Naya Field in the south-eastern direction, with 3D seismic data covering approximately 743 km^2^^10^. Extensive research has been conducted for many years to assess the hydrocarbon potential of the northern part of the Egyptian western desert, as documented in references^4,11–15^. Several writers have explored various facets of the hydrocarbon potential of the Meleiha concession. Several writers have conducted a thorough analysis and assessment of the source rocks found in the Meleiha concession^16,17^. Although many authors evaluated the hydrocarbon reservoirs in the Meleiha concession, including references^4,14,18–20^, none of them have specifically investigated Alam El-Bueib Formation. Alam El-Bueib IIIG (AEB_IIIG) member, in the study area, represents a challenging reservoir due to the lateral and vertical changes in its petrophysical characteristics and net-pay thickness, as reported by Agiba Petroleum Company. The aforementioned issues served as the impetus for the writers to undertake the present investigation. The present work utilizes well logs and core data to identify reservoir levels and evaluate their petrophysical parameters. It also aims at dividing the reservoir into distinct zones that possess similar petrophysical and flow features and to introduce equations that could be used to predict permeability in uncored wells.

## Geologic setting

The North Western Desert (NWD) encompasses several coastal basins that extend along the passive edge of the Mediterranean. These basins formed during the Early Mesozoic period, characterized by the rifting of the Gondwana supercontinent and the subsequent emergence of the Neo-Tethys Ocean^13,21,22^. The pre-rift sedimentary sequence is composed of Paleozoic clastic deposits that are found above the pre-Cambrian basement, as illustrated by the tectono-stratigraphic framework of the region (Fig. 1c)^23^. The process of rifting commenced during the Triassic period and attained its maximum intensity during the Middle Jurassic epoch^13,24,25^. The development of NWD basins occurred inside a sequence of intra-cratonic half grabens oriented in an E-W, ENE-WSW, and NE-SW directions. During the Early Cretaceous epoch, a significant change occurred in the extensional direction, leading to the formation of intricate fault patterns directed in the NE-SW, NW–SE, and E–W directions^15,25,26^.

The basins of the NWD exhibit comparable stratigraphy in terms of basin-fill, consisting of three primary tectono-stratigraphic units that are delineated by unconformity surfaces^23^. The first unit consists of the Paleozoic clastic series, specifically the continental and shallow marine Nubian sandstones and shales. The Ras-Qattara Formation is characterized by the presence of the oldest sediments from the Jurassic period. These sediments primarily consist of non-marine sediments. The sediments are unconformable above the Paleozoic pre-rift facies. The Middle Jurassic period was characterized by the manifestation of subsidence resulting from rift-related events. The subsidence event had a notable impact on the sedimentation process of the Khatatba Formation, characterized by a sequence of transgressions and regressive facies.

The Khatatba Formation is comprised of sandstones and some shales that contain interbedded coal seams. These sedimentary deposits were formed within a deltaic and shallow-water marine setting^27,28^. The initiation of the deposition of the organic-rich facies, Khatatba Formation, which acts as the dominant source rock in the entire north Western Desert, was triggered by the formation of local depocenters adjacent to the major faults^11,12,15,25,29^. The sedimentation process of the Khatatba Formation was marked by intermittent changes in fault offsets, leading to notable variations in both horizontal and vertical facies distribution. Consequently, an intricate assemblage of facies exhibiting progressive progradation and retrogradation was established^15^. The Late Jurassic epoch was distinguished by a sequence of thermal subsidence and extension events, which permitted the deposition of shallow-water carbonates referred to as the Masajid Formation. The deposition of these carbonates exhibits conformity with the underlying Khatatba clastics. The Cimmerian unconformity is superimposed upon the Masajid Carbonates. The aforementioned analyses conducted by researchers^28,30^ provide evidence of a comprehensive phase of inversion, accompanied by tilting, partial erosion, and karstification, within the Jurassic. The sedimentary unit under investigation, AEB Formation, is situated above the unconformity and consists of a combination of shallow marine mixed siliciclastic-carbonate facies. The AEB Formation is geologically dated to the Lower Cretaceous period.

The post-rift unit has two distinct formations. The Bahariya Formation comprises continental and coastal marine clastic sediments that originated during the Upper Cretaceous period and the Abu Roash Formation, which consists of transgressive marine shales and carbonates. The termination of this sedimentation process took place in the Late Cretaceous period, when the African and Eurasian tectonic plates experienced convergence, leading to compression that endured until the Late Eocene epoch^26,31^. A period of tectonic inversion coincided with this phenomenon, which led to the formation of numerous inverted structural patterns that extended as fold belts throughout the entire NWD. Numerous sources^10,25,32^ demonstrate that relatively shallow sedimentary successions with a mix of marine and non-marine Paleogene-Neogene facies follow the Late Cretaceous Abu Roash Formation^10,25,32^.

## Materials and methods

The present work integrates the well logs and core data analysis to evaluate the petrophysical parameters of the AEB_IIIG member reservoir and to identify its different rock types at the field of MWD. Ten wells with complete set of essential logs including GR, Shallow, Mid, and Deep Resistivity, Neutron, Sonic, and Density logs, were provided to identify and evaluate the petrophysical characteristics of AEB_IIIG Member of Alem El-Bueib Formation (Fig. 1b). Schlumberger TechLog and Interactive Petrophysics (IP) software were used to analyze the logs and determine the reservoir potential of the AEB_IIIG member in the examined wells. The M–N cross plot utilizes the density, neutron, and sonic logs to discern the mineral compositions^33^. The terms M and N are defined as follows:

1 $${\text{M}} = \frac{{{\Delta t}_{{{\text{fl}}}} - {\Delta t}_{{{\text{log}}}} }}{{{\uprho }_{{\text{b}}} - {\uprho }_{{{\text{fl}}}} }} \times 0.01$$

2 $${\text{N}} = \frac{{\emptyset_{{{\text{Nfl}}}} - \emptyset_{{{\text{Nlog}}}} }}{{{\uprho }_{{\text{b}}} - {\uprho }_{{{\text{fl}}}} }}$$

Reservoir gross thickness, shale volume, total and effective porosity, water resistivity and saturation, net-pay thickness, and hydrocarbon saturation are among the inferred reservoir metrics. Standard interpretation techniques of^34,35^ were utilized to conduct the wireline logging analyses using the Eqs. (3), (4), (5), and (6) as follows:

3 $${\text{Vsh}} = \left( {GR log - GR min} \right)/\left( {GR max - GR min} \right)$$

4 $$\Phi eff = \frac{{\left( {\Phi NC + \Phi DC } \right)}}{2 }$$

5 $$\frac{1}{{R}_{t}}=\frac{{S}_{w}^{n}}{{F}_{0}}\left[\frac{1}{{R}_{w}}+\frac{{V}_{Q}{Q}_{V}}{{S}_{wt}}\left(\frac{1}{{R}_{cw}}-\frac{1}{{R}_{w}}\right)\right]$$

6 $${S}_{hr}=1-{S}_{w}$$

where ${\text{Vsh}}$, shale volume, $\Phi eff$, Effective porosity, $\Phi NC$, corrected Neutron, $\Phi DC$, corrected Density, R_t_, Resistivity of the formation; R_w_, Resistivity of the formation water; R_cw_, resistivity of the bound water; Q_v_, Effective concentration of clay counterions; V_Q_, clay-water volume; F, Formation resistivity factor.

The vertical and lateral distribution of the calculated parameters of AEB_IIIG level are then achieved through isoparametric contour maps and the lithosaturation panels.

A set of 129 thin sections was carefully prepared from the conventional and side-wall core plugs obtained from the AEB_IIIG clastic interval in MWD-9 Well. The thin slices were created at the EPRI (Egyptian Petroleum Research Institute)-CORE Analysis Lab, which is situated in Egypt. The thin section preparation procedure involved the application of vacuum impregnation using blue-dyed resin. This measure was undertaken in order to improve the detection and examination of porosity^36^. The thin sections were then looked at with a polarized microscope to find out what their main parts were and how many of them there were. This was done using the established point-counting method developed by^37^. The lithological composition of the studied thin sections match that derived from the analysis of wireline logs.

Numerous methodologies have been suggested to effectively conduct rock typing. Certain methodologies rely on the geological characteristics of reservoirs, one of which being the Lucia approach^38^. Furthermore, many petrophysical techniques have been established based on specific reservoir characteristics, including porosity (*ϕ*) and permeability (K). Moreover, many empirical and theoretical measures have been devised to facilitate the process of petrophysical rock typing (PRT). Rocks of different rock types demonstrate commonalities in both their static features and dynamic qualities, which are linked to the behavior of fluid flow. The aforementioned commonalities are commonly known as petrophysical static rock typing (PSRT) and petrophysical dynamic rock typing (PDRT)^39^. In scholarly discourse, the terminology of PRTs and hydraulic flow units (HFU) has been employed interchangeably. In their extensive analysis^40^, thoroughly examined a range of rock typing methodologies that have been employed in both scholarly literature and industrial applications^40^. The flow zone indicator (FZI) is a modified version of the Kozeny-Carman equation that quantifies the correlation between micro-scale characteristics, including pore shape, size, pore throat radius, and aspect ratio, and macro-scale qualities like porosity and permeability. This correlation is expressed in Eq. (7),

7 $$k=\phi \frac{{r}_{mf}2}{{F}_{s}t}$$

where r_mf_ represents the average radius of the hydraulic unit, F_s_ denotes the shape factor, and t represents the hydraulic tortuosity, defined as the ratio of the actual length to the straight length (L_a_/L).

Theoretical investigations can establish a correlation between micro-scale qualities and macro-scale factors that are easy to measure, such as porosity and permeability obtained through routine core analysis (RCAL)^41^. To determine the FZI by this approach, it is necessary to compute the reservoir quality index (RQI) and the normalized porosity ($\phi$_z_) using Eqs. (8) and (9), respectively. Lastly, Eqs. (8 and 9) can be employed for the computation of the FZI as in Eq. (10).

8 $${\text{RQI}} = 0.0314\sqrt {K/\phi }$$

9 $$\phi_{z} = \phi /(1 - \phi )$$

10 $${\text{FZI }} = {\text{ RQI}}/\phi_{z}$$

where k is permeability (in millidarcy), and ∅ is porosity (in volume fraction).

Numerous scholars have endeavored to change the FZI by either incorporating or diminishing the quantity of parameters necessary for computing the index^42–45^. Rock typing is an essential tool for the estimation of reservoir parameters in areas where obtaining expensive core samples is not feasible. Therefore, several theoretical and empirical models have been presented to estimate various properties, with the most significant ones being permeability (K) and porosity ($\phi$). The Winland empirical equation is frequently used as a rock type index, in conjunction with the FZI, by considering the pore throat radius at a mercury saturation level of 35%.

Conventional core analysis on 140 cylindrical core plugs from the AEB-G interval in Meleiha-West-Deep-09 borehole. The conventional core analysis measures porosity, permeability, grain density, and water saturation. Porosity and permeability data were obtained using helium porosimeters and nitrogen permeameters, respectively. The FZI, normalized porosity index (NPI), and RQI were computed as functions of porosity and permeability^46^.

Furthermore, using the Winland formula^47–50^, the average effective pore throat radius (R35) values were estimated from the observed core porosity and permeability values as follows:

11 $${\text{Log }}\left( {R35} \right) \, = \, 0.732 + 0.588\log \left( K \right) \, = \, 0.864\log \left( \Phi \right)$$

The pore aperture radius denoted as R35 is the radius at which the 35th percentile of mercury saturation is observed. K denotes the permeability, measured in millidarcies (mD), while ∅ represents the porosity, expressed as a percentage. According to^51^, the most optimal outcome for predicting permeability in sandstones was observed to be R25. According to the findings of^52^, it has been proposed that the pore throat size R50 exhibits the highest level of reliability when utilized for the prediction of permeability in carbonate formations. The distinction of five petrophysical flow units with varying reservoir performance is delineated by ranges of R35, as stated by^53,54^.

The term “mega-porous” is used to describe rock units that have pore throat radii larger than 10 μm. Macro-porous describes pore throat radii ranging from 2.5 to 10 μm. Meso-porous refers to materials that have pore throat radii ranging from 0.5 to 2.5 μm. Rock units with small pores, ranging in size from 0.2 to 0.5 μm, are called micro-porous, while the term “nanopores” refers to rock units that have pore throat radii less than 0.2 μm.

The utilization of the stratigraphic modified Lorenz plot (SML) is a significant technique for subdividing the reservoir sequence into Hydraulic Flow Units (HFUs). The significance of the SML approach stems from its direct correlation with the reservoir's storage capacity. The estimation of the flow and storage capacity of the analyzed sequence is conducted in an accumulative way. The efficacy of the SML approach in partitioning the reservoir into HFUs can be inferred from the slope range of each HFU line segment^55^. The stratigraphic modified Lorenz (SML) plot has been extensively utilized by numerous researchers to partition the reservoir into distinct hydraulic flow units (HFUs). These HFUs are characterized as either non-conductive (such as tight, barrier, or seal), conductive, or super-conductive zones^50^. Numerous authors have applied and validated this concept in the past twenty years (e.g.,^56–66^).

## Results

### Petrophysical analysis

**Figure 2:**
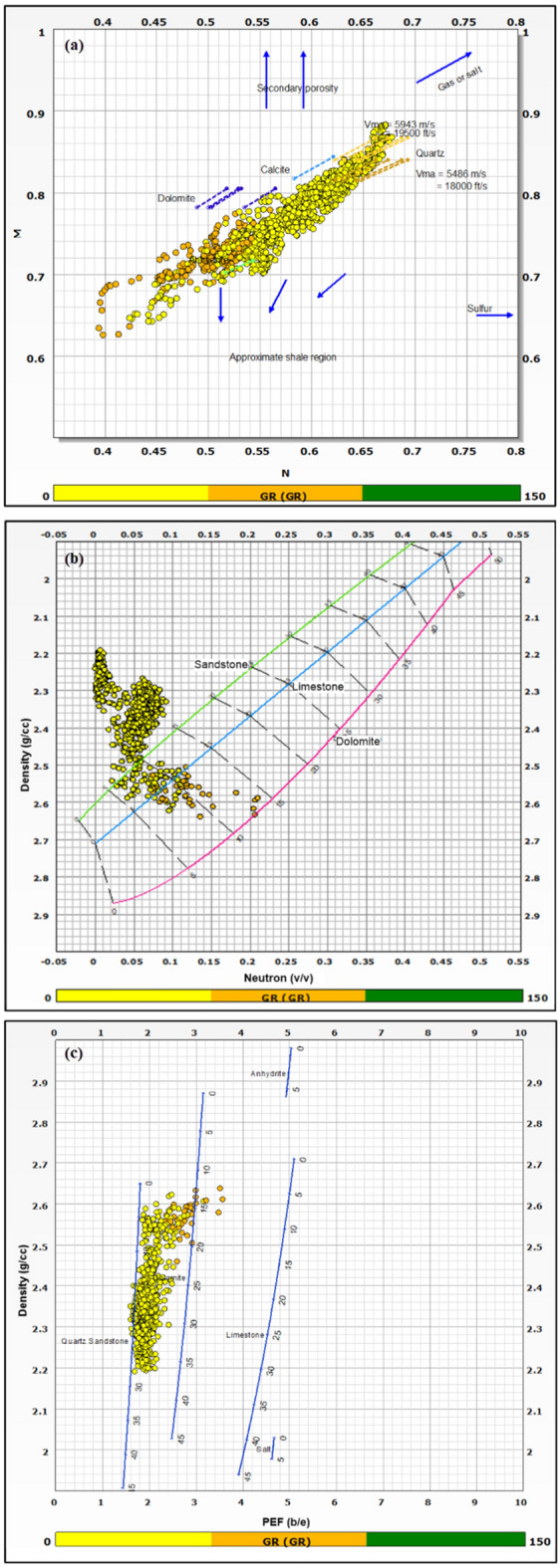
(**a**) MN cross plot showing the mineralogical composition of the AEB_IIIG reservoir, (**b**) Neutron-density cross plot depicting the dominant lithology in the AEB_IIIG reservoir, and (**c**) PEF-density cross plot depicting the prevalent lithology within the AEB_IIIG reservoir.

**Figure 3:**
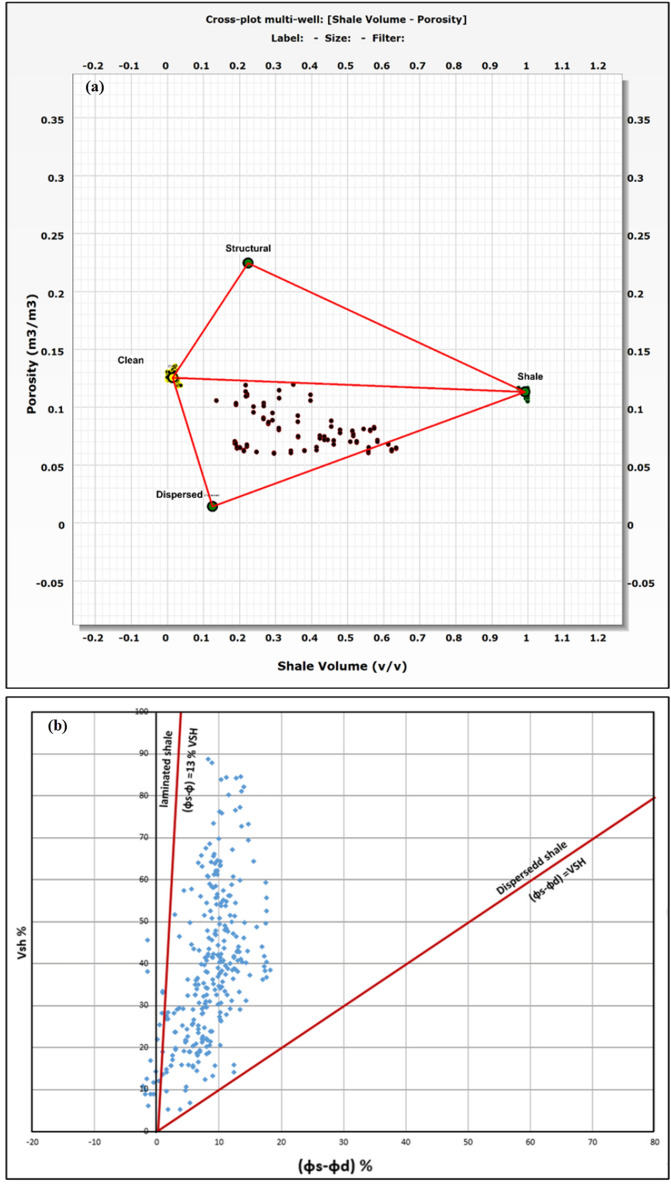
(**a**) Thomas-Stieber cross plot depicting that the dominant shale type is dispersed, and (**b**) Shale type cross plot demonstrating dispersed shale is the dominant shale type.

**Figure 4:**
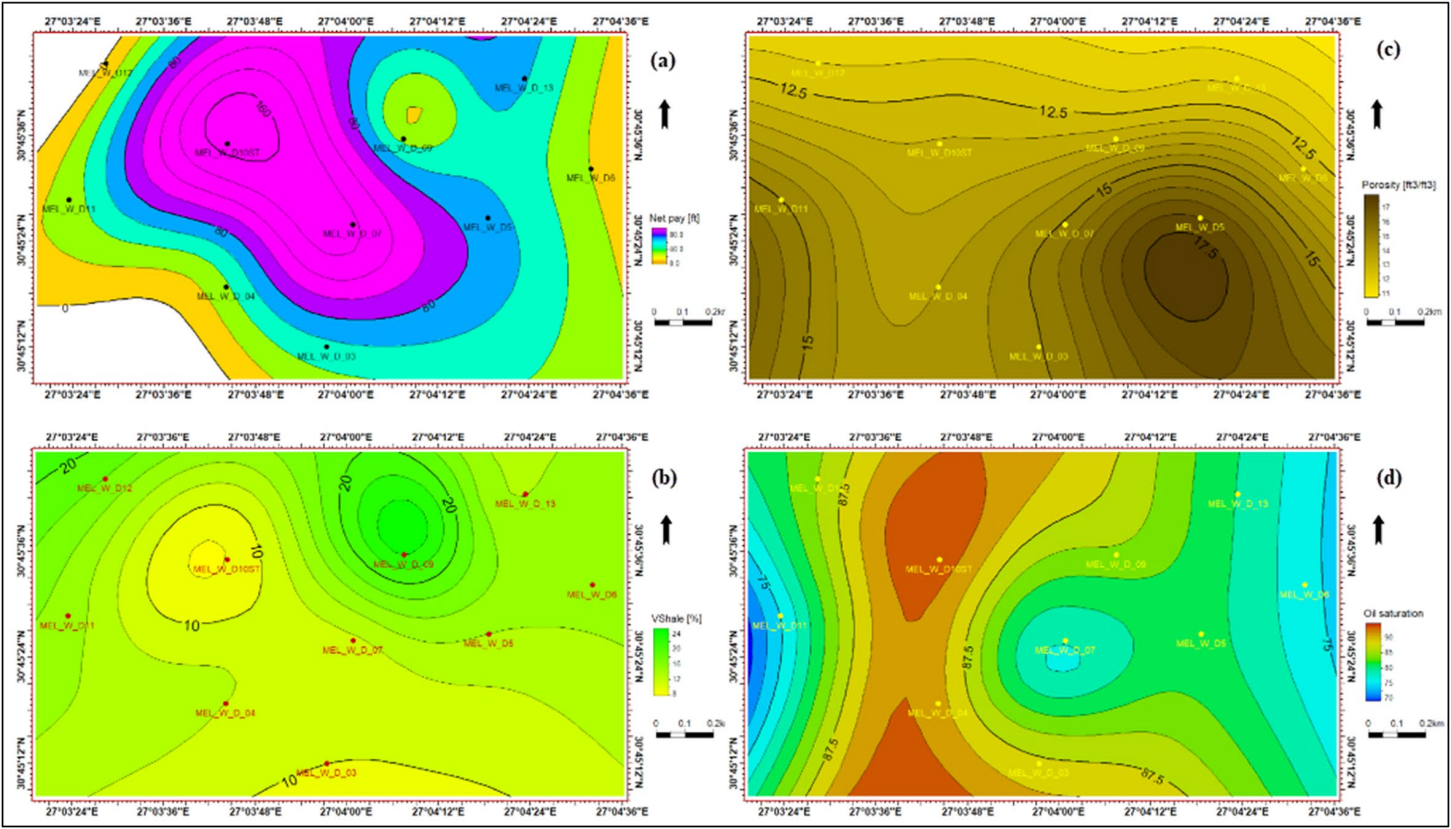
(**a**) The net-pay thickness map illustrating the distribution of the net pay thickness over the area, (**b**) A contour map illustrating the distribution of the shale volume over the area, (**c**) A contour map illustrating the distribution of the effective porosity over the area, and (**d**) A contour map illustrating the distribution of the hydrocarbon saturation over the area.

**Figure 5:**
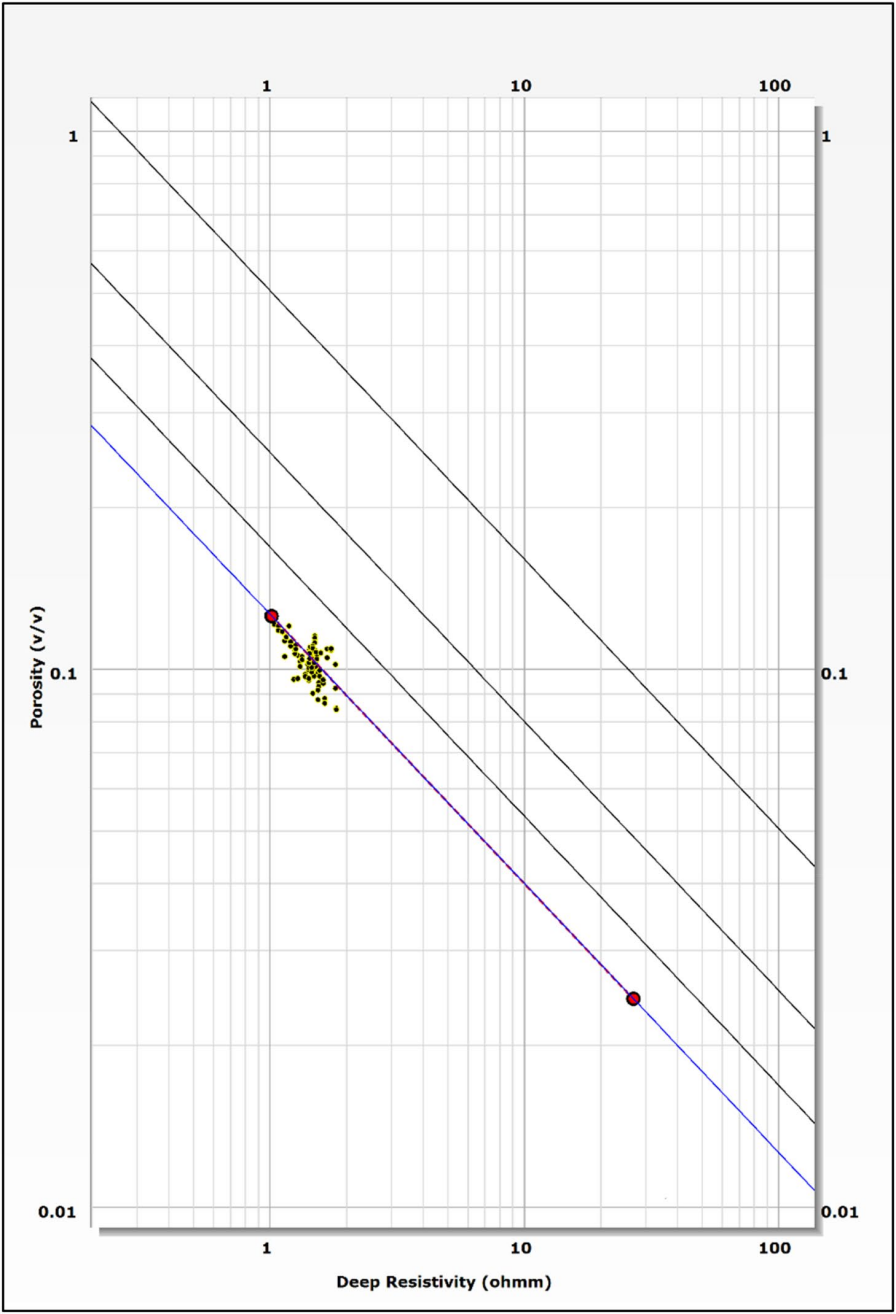
Pickett’s plot utilized for water resistivity calculations.

**Figure 6:**
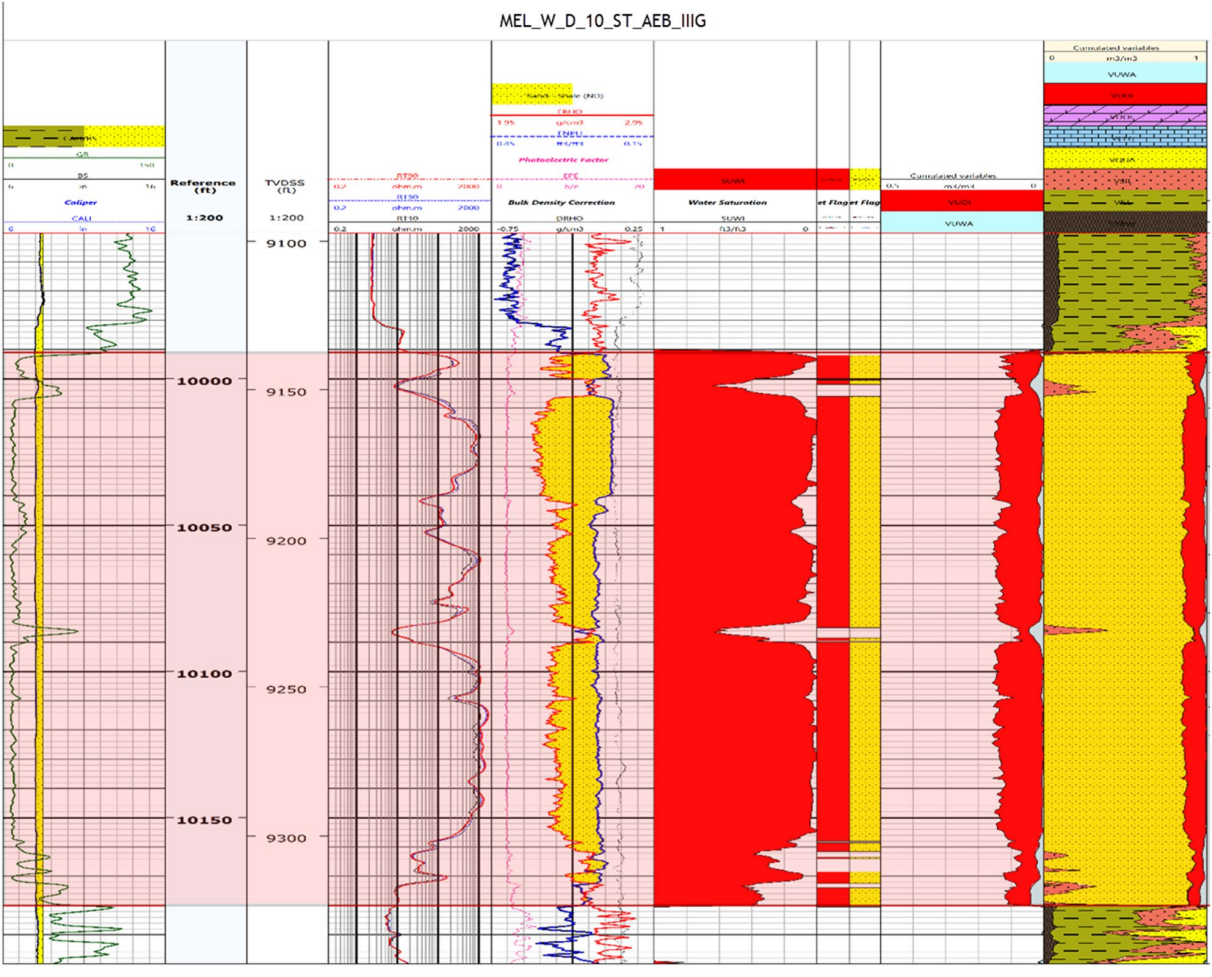
A lithosaturation panel illustrating the vertical distribution of the petrophysical parameters and lithology.

**Figure 7:**
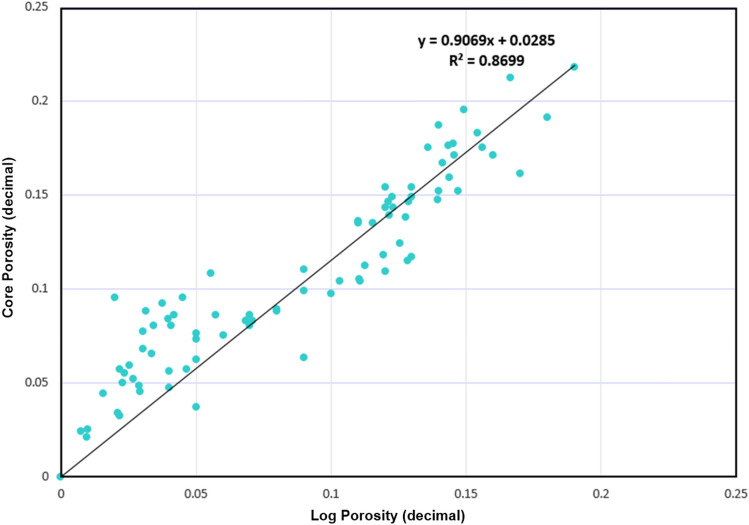
The correlation between log-derived and core-derived porosities shows a good match with R^2^ equals 0.8699.

The identification and evaluation of the reservoir interval of the AEB_IIIG member were conducted by analyzing the geophysical records of ten wells that penetrated the said reservoir interval. The utilization of log responses and lithological identification charts facilitated the process of characterizing the mineralogical and lithological composition of the reservoir^67^ and^68^. The utilization of the MN cross plot facilitated the differentiation between shale and sand intervals (Fig. 2a). Neutron-density and (photoelectric factor) PEF-density cross plots provide evidence that the reservoir interval of AEB_IIIG consists of quartz sandstone (Fig. 2b and c respectively). Based on the analysis of logging data and graphical representations, a definitive inference can be drawn that AEB_IIIG exhibits the characteristics of a sandstone reservoir. The examination of the thin sections acquired from core samples validates the lithological composition inferred from the wireline logs. The identification of the shale type was accomplished by the utilization of Thomas-Steiber and shale type cross plots (Fig. 3a and b respectively). There is a significant variation in the net pay thickness of the AEB_IIIG reservoir, ranging from 2.5 ft in the MEL_W_D12 well to 168 ft in the MEL_W_D10ST well (Fig. 4a). The shale-content values obtained from the calculations exhibit a range spanning from 8 to 25% (Fig. 4b). The neutron and density logs provided were employed in the computation of the effective porosity. The estimated effective porosity for the AEB_IIIG reservoir ranges from 12 to 17% (Fig. 4c). The formation-water resistivity in the interval of interest was determined using Pickett's plot, yielding a value of 0.016 Ohm.m (Fig. 5). The utilization of water resistivity and deep resistivity logs, in conjunction with the determination of effective porosity, facilitated the estimation of hydrocarbon saturation inside the AEB_IIIG reservoir. The saturation of hydrocarbons varies between 72 and 92% (Fig. 4d). The vertical distribution of all computed petrophysical parameters pertaining to the AEB_IIIG reservoir is illustrated in the lithosaturation plot (Fig. 6). Figure 6 displays the reservoir's level, characterized by the presence of sandstone indicated by the low gamma ray (first track) and high hydrocarbon saturation indicated by the high resistivity (fourth track). A correlation between the log-derived and core-derived porosity would enhance the reliability of the study^69^. The estimated log-derived porosity has a good correlation coefficient with the corrected core-derived porosity, with R^2^ = 0.869 (Fig. 7). Table 1 presents the mean values of the petrophysical parameters pertaining to the AEB_IIIG reservoir across all wells examined in the study.

**Table 1:** The average values of the calculated petrophysical parameters of the AEB-3G reservoir in all studied wells in the area.

Well name	Net pay thickness (ft)	Effective porosity (%)	Shale volume (%)	Hydrocarbon saturation (%)
MEL_W_D3	55.5	15	10	88
MEL_W_D4	37	14	12	91
MEL_W_D5	68	17	14	81
MEL_W_D6	19.5	13	15	76
MEL_W_D7	142	15	12	78
MEL_W_D9	26.5	13	25	85
MEL_W_D10ST	168	13	8	94
MEL_W_D11	30	15	15	75
MEL_W_D12	2.5	12	18	85
MEL_W_D13	70	12	15	82

### Petrography and microfacies

**Figure 8:**
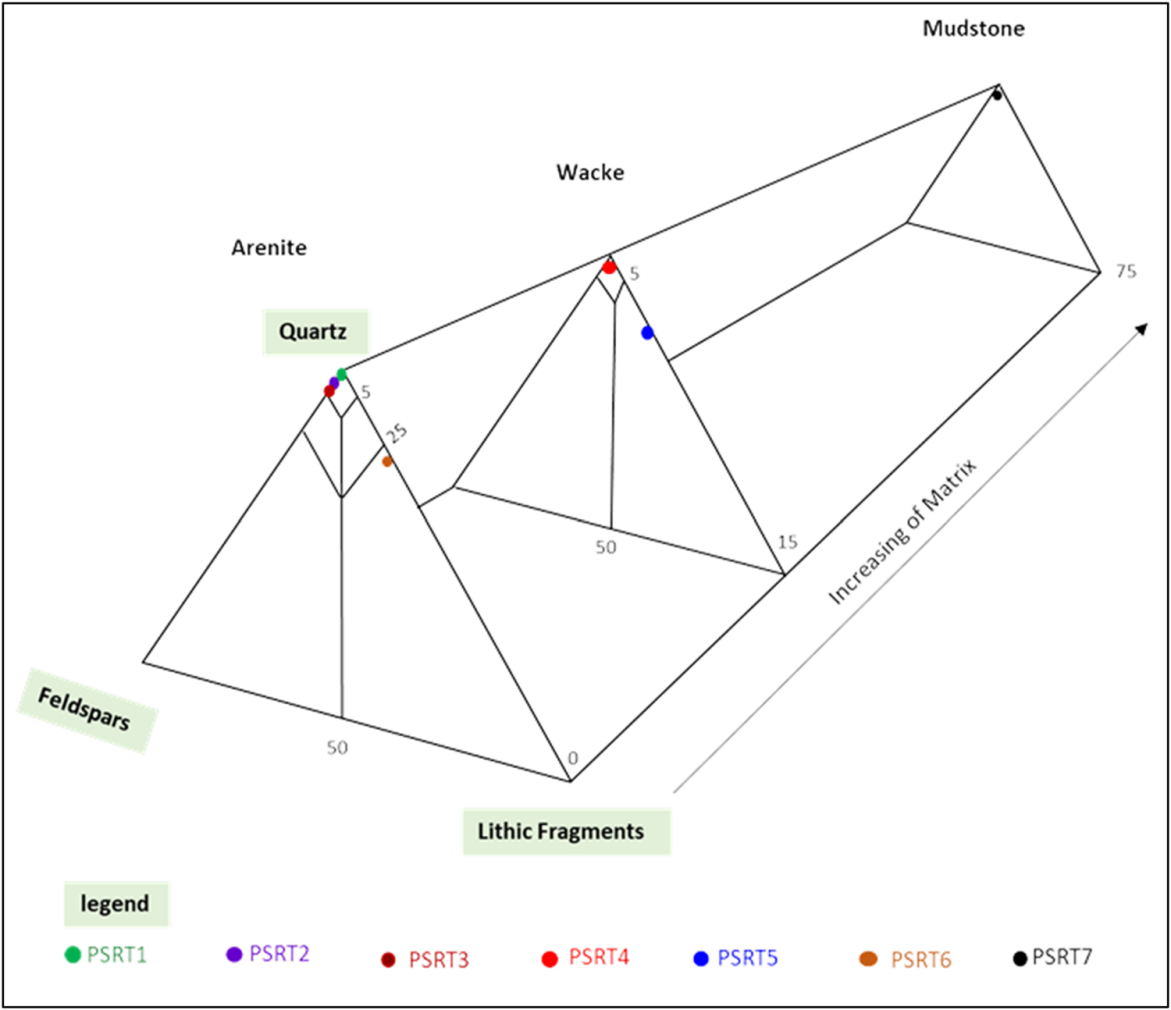
Quartz-feldspar-lithic fragments (QFL) ternary plot showing the modal classification of the AEB sandstones^63^.

**Figure 9:**
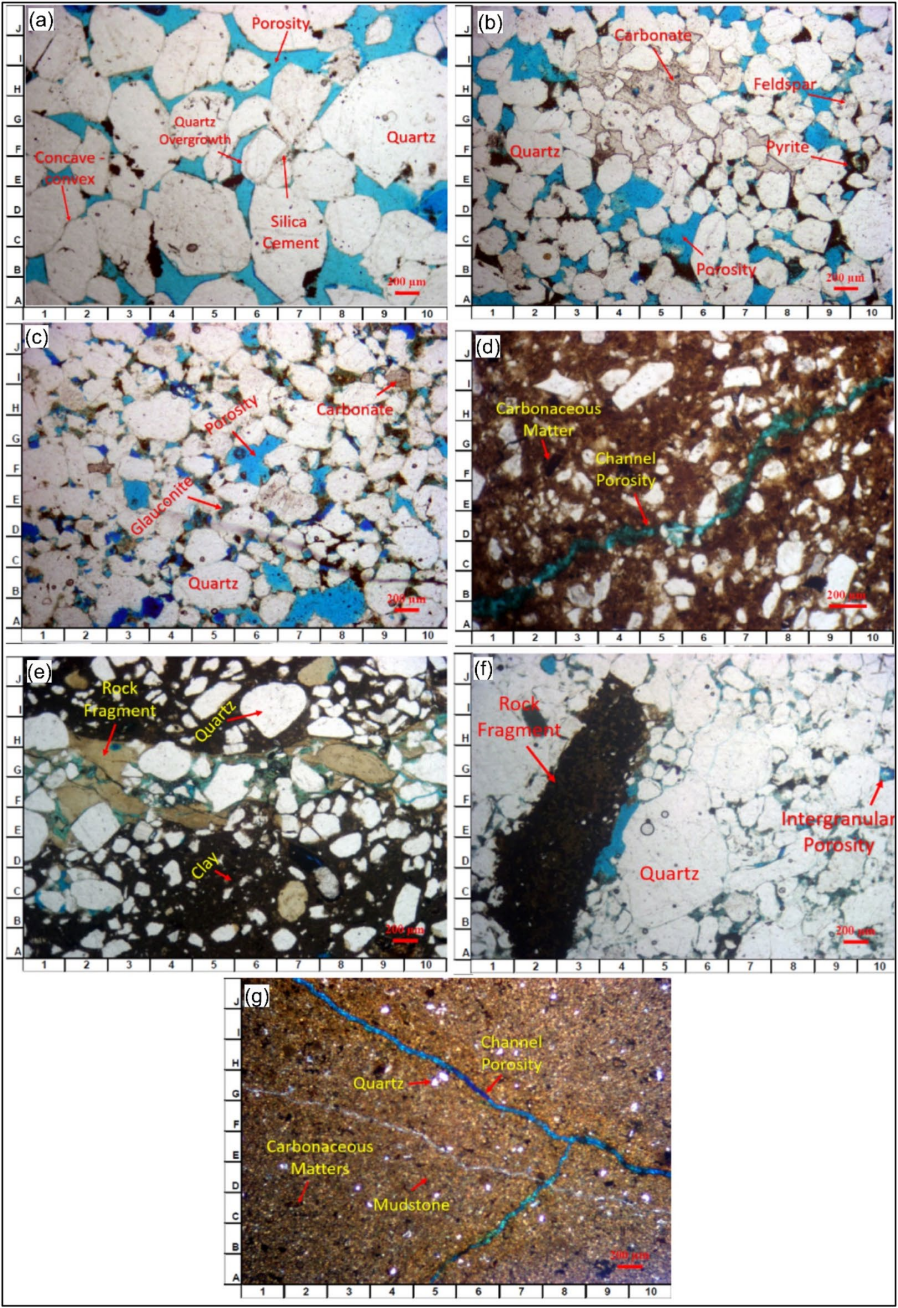
Thin-section microphotographs depicting the textural and mineralogical characteristics of the AEB_IIIG sandstone (**a**) Quartz arenite (MF1), (**b**) Calcareous quartz arenite (MF2), (**c**) Glauconitic quartz arenite (MF3), (**d**) Quartz wacke (MF4), (**e**) Lithic wacke (MF5), (**f**) Lithic arenite (MF6), and (**g**) Mudstone (MF7).

The AEB_IIIG sandstones exhibit a grain-supported texture characterized by a relatively low proportion of matrix material. The core samples obtained from MEL WEST DEEP-9 well have been categorized based on the sandstone classification methodology (Fig. 8)^70^. The AEB_IIIG sandstones were classified into seven distinct sandstone microfacies, taking into consideration their primary composition and the relative proportions of quartz, feldspars, and rock fragments present within them. The identified microfacies (MF) consist of the following: (1) Quartz arenite (MF1), (2) Calcareous quartz arenite (MF2), (3) Glauconitic quartz arenite (MF3), (4) Quartz wacke (MF4), (5) Lithic wacke (MF5), (6) Lithic arenite (MF6), and (7) Mudstone (MF7). An example of each microfacies is illustrated by a thin section (Fig. 9). The petrographic modal composition of the AEB_IIIG sandstone microfacies is tabulated in Table 2.

**Table 2:** The petrographic modal composition of the AEB_IIIG sandstone microfacies.

Components (%)	Quartz	Lithic Fragments	Feldspars	Glauconite	Quartz Cement	Carbonate Cement	Pyrite	Clays	Point Count Porosity	Total
Quartz arenite (MF1)										
Min	50	0	0	0	0	0	4.4	0.8	5.2	
Max	70	0	3.6	11.2	6.4	0	14.4	9	22	
Avg	64	0	1.4	3.9	3.5	0	7.2	4.3	15.7	100.0
Calcareous quartz arenite (MF2)										
Min	45	0	0	0	0	7	2.4	3	1.8	
Max	60.8	3.2	6.04	6.4	8.4	12	7	4.8	21.6	
Avg	56.3	1.3	5	3.8	3.5	8.5	4	3.5	14.1	100.0
Glauconitic quartz arenite (MF3)										
Min	42	0	3	10	6	0	3	5	18	
Max	45	0	5	14	8	3	6	6.6	20	
Avg	46.2	0	3.7	12	7	1.4	5	5.7	19	100.0
Quartz wacke (MF4)										
Min	33.2	0	0	0	0	0	2	46	9.2	
Max	37	2	0	0	2	0	5.2	52.4	12	
Avg	35.6	2	0	0	1	0	3	48	10.4	100.0
Lithic wacke (MF5)										
Min	34.4	12.8	0	1	0	0	7	27.2	2.4	
Max	41	15	0	5	0.8	3	12.4	34.4	5	
Avg	37.5	13.9	0	3	0.4	1.7	9.1	30.6	3.8	100.0
Lithic arenite (MF6)										
Min	51.6	19.2	0	0	7.2	0	2	3.8	5	
Max	60	21	0	1.2	14.4	1	5	4.8	8	
Avg	56.5	19.4	0	0.6	9.8	0.5	2.7	4.5	6	100.0
Mudstone (MF7)										
Min	4.4	0	0	0	0	0	0	87	3	
Max	7.3	2	0	1.2	0	0	0	91.2	4.4	
Avg	6.2	1	0	0.8	0	0	0	88.6	3.4	100.0

1. Quartz arenite (MF1) (9948.95 ft. depth)
  Quartz arenite is the best microfacies in the reservoir. The detrital framework grains of MF1 are dominated by Subrounded to Subangular, medium –very coarse quartz grains, moderately sorted (average = 60%). Matrix content is very low, while other detrital and authigenic mineral phases are relatively rare. MF1 is moderately to strongly compact where the grain-to-grain contacts are mainly sutured and concavo-convex. Quartz overgrowth and silica cement are the most common authigenic mineral phases in MF1. Large intergranular pores are observed and have good connectivity, and therefore, MF1 sandstones have a well-connected pore network (Fig. 9a).
2. Calcareous quartz arenite (MF2) (10,001.45 ft. depth).
  (MF2) sandstones comprise a carbonate-rich quartz arenite. Quartz grains are rounded to sub-angular, Medium to Fine Occasionally Coarse grains, moderately sorted. Matrix content is low. Feldspars are represented by slightly dissolved K-feldspars (Fig. 9b). Carbonate cements (average = 8.5%) are represented by emerging patches of pore-filling Calcite. Both Primary porosity (intergranular porosity) and secondary porosity (leaching of clay matrix and dissolution) are represented. MF2 pore network is partly blocked where the size of most pore throats is smaller than that in MF1.
3. Glauconitic quartz arenite (MF3) (10,003.45 ft. depth).
  MF3 sandstones comprise a Glauconite -rich quartz arenite, is made of rounded to sub- angular, fine—coarse grains, poorly sorted quartz grains, partly dissolved K-feldspars, Detrital clay matrix is existing, and the intergranular pores are mainly filled with Glauconite (average = 12%). Both Primary porosity (intergranular porosity) and secondary porosity (leaching of clay matrix and dissolution) are represented (Fig. 9c).
4. Quartz wacke (MF4) (10,017.65 ft. depth)
  Quartz Wacke (MF4) is made of Quartz grains which is coated with clays. Quartz grains are fine to medium -grained, moderately sorted, subangular to angular. Feldspars and rock fragments are rarely observed. Clays in MF4 occur as massive loose aggregates and occasionally form thin coatings around the detrital framework grains. Clay ferruginous and dolomite cement are represented. The porosity is mainly secondary (channel porosity) (Fig. 9d).
5. Lithic wacke (MF5) (10,080.9 ft. depth)
  Lithic wacke (MF5) is made of a Quartz grain which is small enough, rich matrix and coated with clays. Quartz grains are sub-angular to rounded, very fine to Very coarse grains, poorly sorted. Clay, ferruginous and dolomite cement are represented (Fig. 9e). Intragranular porosity is reported.
6. Lithic arenite (MF6) (10,061.1 ft. depth)
  Lithic Arenite (MF6) framework is made of Quartz grains (average = 56.5%) and rich rock fragments. Quartz grains are sub-angular to sub-rounded, fine to Very coarse grains, poorly sorted. Cementation, compaction, and quartz overgrowth are observed. Clay, celiac cement is represented. Intergranular porosity is reported due to dissolution (Fig. 9f).
7. Mudstone (MF7) (10,084.08 ft. depth)
  Mudstone (MF7) is mainly composed of mudstone groundmass, with traces of quartz grains. These quartz grains ranges from very fine to fine and is distributed as scattered grains within mudstone groundmass. Some carbonaceous matters are scattered in the groundmass and some microfossils are also observed in the mud groundmass. The porosity is mainly secondary (channel porosity) (Fig. 9g).

The establishment of a correlation between the counted porosity and the lithological composition results in the subdivision of the five microfacies into seven distinct units. The Quartz Arenite can be subdivided into three distinct units, each characterized by varying porosity levels of 22%, 16%, and 14% respectively.

### Rock typing

**Figure 10:**
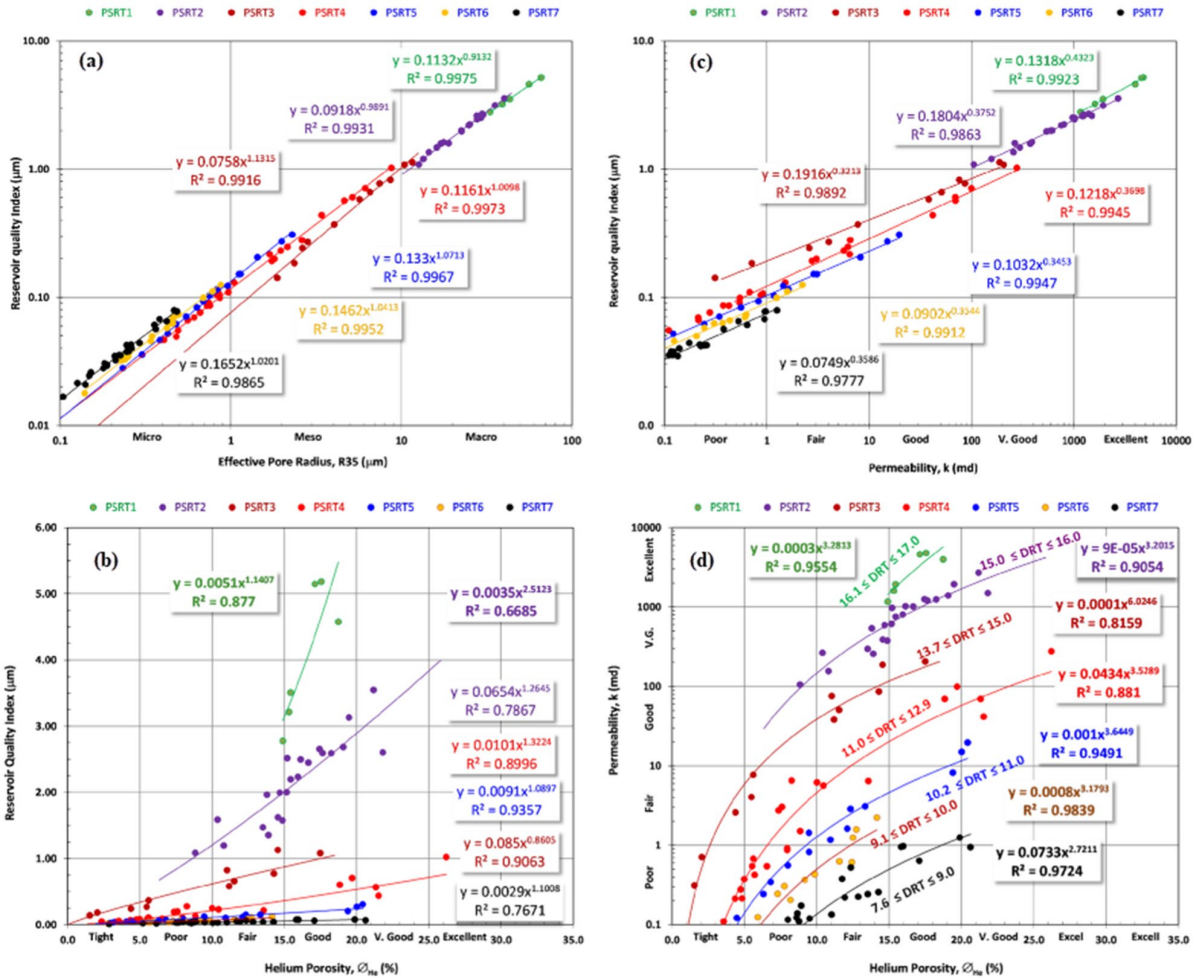
(**a**) The relationship between R35 and RQI for the seven rock types illustrating equations and R^2^ for each rock type, (**b**) The relationship between helium porosity and RQI for the seven rock types illustrating equations and R^2^ for each rock type, (**c**) The relationship between permeability and RQI for the seven rock types illustrating equations and R^2^ for each rock type, and (**d**) The relationship between helium porosity and permeability for the seven rock types illustrating equations and R^2^ for each rock type.

By employing RCAL and employing several rock-typing methodologies such as RQI and FZI, a total of seven petrophysical static rock types were discerned, denoted as psrt1, psrt2, psrt3, psrt4, psrt5, psrt6, and psrt7. Table 3 presents a comprehensive tabulation of the primary petrophysical parameters associated with each distinct rock type. There is significant change in the petrophysical parameters and features of the pore system among the rock types examined in the AEB_IIIG reservoir. The recorded data from the RCA indicate that there are higher levels of horizontal permeability in psrt1, with an average measurement of 3010.37 millidarcies (mD). The helium-porosity values are higher in psrt1 compared to the other PSRTs. Nevertheless, the average values of the effective porosity observed in the AEB_IIIG psrts exhibit minimal variation, ranging from 9 to 17%. Hence, the observed significant heterogeneity in permeability values among the examined sedimentary facies can be attributed to disparities in pore structure and geometry, rather than changes in pore volume. The RQI data indicate that psrt1, psrt2, and psrt3 possess a favorable reservoir quality, with an average RQI of around 4.07, 2.16 and 0.6 μm, respectively. In contrast, the remaining psrts have average RQI values below 0.2 μm. The FZI serves as a representation of the presence of fluid flow zones of high quality in psrt1, psrt2, and psrt3, where the average FZI around 20, 11 and 6 μm respectively. In contrast, the remaining psrts have average FZI values below 1 μm. Additionally, it is important to acknowledge that R35 average values greater than 6 μm are only found in psrt1, psrt2 and psrt3, whilst the other psrts demonstrate typical R35 values below 6 μm. The RQI demonstrates a notable degree of association, as evidenced by the considerably high R^2^ values, with respect to various psrts. This correlation is particularly strong in reference to R35, helium porosity, and permeability. Figure 10a depicts the link between the RQI and R35. Conversely, Fig. 10b and c illustrate the correlation between RQI and helium porosity, as well as permeability, respectively. There is a significant correlation coefficient observed between helium porosity and permeability across all psrts (Fig. 10d). The equations derived from the correlation analysis between helium porosity and permeability (Fig. 10d and Table 4) have the potential to be utilized for estimating permeability in uncored intervals of the AEB_IIIG reservoir in nearby wells within the field.

**Table 3:** Petrophysical core data and reservoir quality parameters for the AEB-3G reservoir petrophysical static rock types (PSRTs).

PSRT	∅	∅Z	k_H_	k_V_	RQI	FZI	R_35_
No	%	%	md	md	μm	μm	μm
PSRT1							
Min	15	18	1164.17	19.81	2.77	15.84	33.19
Max	19	23	4785.89	5036.95	5.18	24.92	66.15
Average	17	20	3010.37	2604.53	4.07	20.32	50.66
PSRT2							
Min	9	10	105.22	8.04	1.08	8.38	12.69
Max	22	28	2703.31	2562.90	3.55	14.02	40.21
Average	16	19	881.10	707.12	2.16	11.46	24.36
PSRT3							
Min	2	2	0.31	0.01	0.14	4.61	1.87
Max	18	21	206.52	712.41	1.13	9.05	11.58
Average	9	10	60.06	98.53	0.57	6.05	5.83
PSRT4							
Min	2	2	0.06	0.00	0.05	1.20	0.41
Max	26	36	277.01	20.92	1.02	3.09	8.76
Average	10	11	23.79	2.49	0.23	1.86	2.00
PSRT5							
Min	3	3	0.02	0.00	0.03	0.84	0.23
Max	20	26	19.65	3.16	0.31	1.20	2.29
Average	10	12	3.46	0.69	0.12	1.00	0.89
PSRT6							
Min	3	3	0.01	0.00	0.02	0.48	0.14
Max	14	16	2.23	0.41	0.12	0.76	0.88
Average	9	10	0.52	0.11	0.06	0.63	0.43
PSRT7							
Min	3	3	0.00	0.00	0.01	0.23	0.05
Max	21	26	1.25	3.64	0.08	0.46	0.48
Average	11	12	0.28	0.26	0.04	0.33	0.25

**Table 4:** The derived equations for the AEB-3G reservoir petrophysical static rock types (PSRTs) with their reflection coefficient (R^2^) (Y: Permeability, X: Porosity).

Rock type	Equation	R^2^
PSRT 1	Y = 0.0003 X^3.2813^	0.9554
PSRT 2	Y = 0.0009X^3.2015^	0.9054
PSRT 3	Y = 0.0001 X^6.0246^	0.8159
PSRT 4	Y = 0.0434 X^3.5289^	0.881
PSRT 5	Y = 0.001 X^3.6649^	0.9491
PSRT 6	Y = 0.0008 X^3.1793^	0.9839
PSRT 7	Y = 0.0733 X^2.7211^	0.9724

**Figure 11:**
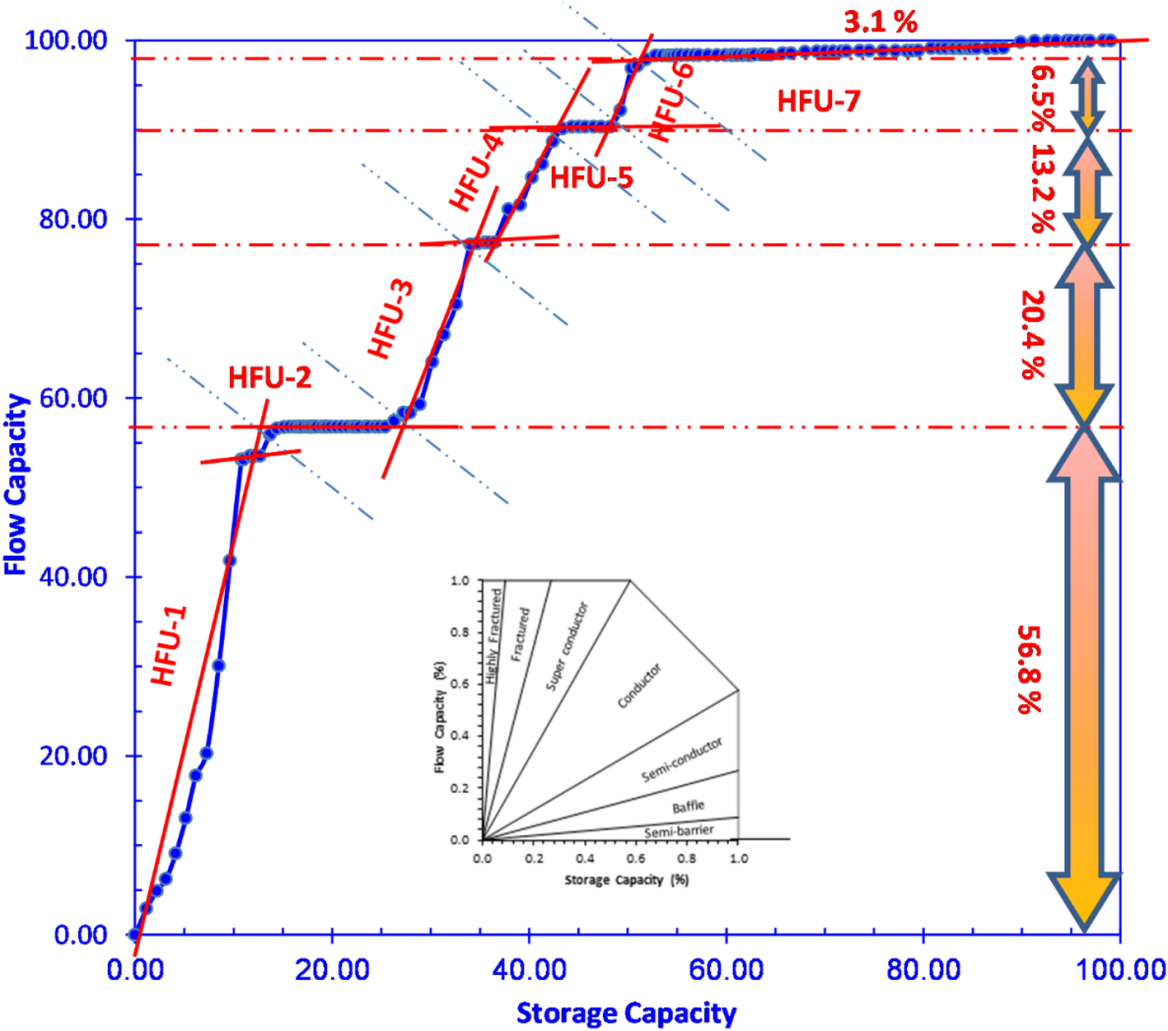
The stratigraphy modified Lorenz plot illustrating the relationship between storage capacity and flow capacity for the seven rock types.

The SML plot illustrates the classification of the reservoir under study into seven HFUs (Fig. 11). The units exhibit varying storage and flow capabilities, with HFU1 demonstrating a notably high capacity, while HFU7 showcases a relatively low capacity. Those units are comparable with those identified using other techniques including petrography and petrophysical data.

## Discussion

While previous studies have examined the source and reservoir rocks in the Meleiha area (e. g.,^4,14,16–20^), this study is the first to specifically focus on the evaluation, characterization and typing of the AEB Formation reservoir in the MWD oil field using well logs and core data. The objective of the investigation described in this paper was to categorize and characterize the reservoir rocks in the MWD field by analyzing their petrographical and petrophysical features. The findings derived from the investigation offer significant insights into the diversity and potential fluid flow dynamics inside the reservoir. The utilization of log-derived lithological identification charts facilitated the identification of the mineralogical and lithological composition of the AEB_IIIG reservoir. The thin sections of the core samples acquired from the same interval of the AEB_IIIG reservoir validated the composition inferred from the logs. The predicted porosity calculated using log data has a strong correlation coefficient with the corrected core porosity, with an R^2^ value of 0.869. The analysis conducted in this study has identified the existence of seven distinct rock types within the surveyed area. These rock types were generally categorized based on their porosity, permeability, and lithology. The thin sections of the examined samples revealed the existence of seven distinct rock types: (1) Quartz arenite (MF1), (2) Calcareous quartz arenite (MF2), (3) Glauconitic quartz arenite (MF3), (4) Quartz wacke (MF4), (5) Lithic wacke (MF5), (6) Lithic arenite (MF6), and (7) Mudstone (MF7). Several writers (e.g.^55,57,58,71–80^) have employed various rock typing methodologies. In the present study, several rock typing techniques were used and compared to identify seven distinct rock types, which have been labeled as psrt1, psrt2, psrt3, psrt4, psrt5, psrt6, and psrt7. The seven rock types correspond to the seven MF obtained from the thin sections. Also, the seven HFUs derived from the SML plot could correspond to the seven rock types obtained from the other employed techniques. Since identifying and locating the different flow units help decision-makers in the field of petroleum industry, the findings of this work demonstrate the applicability of the methodologies used and contribute to future reservoir evaluation and field development.

## Conclusion

The conclusions drawn from the present investigation are as follows: The AEB_IIIG reservoir exhibits significant variations in net pay thickness as well as petrophysical properties such as shale volume, effective porosity, and hydrocarbon saturation. The calculated estimates for shale content demonstrate a variability ranging from 8 to 25%. The effective porosity has a range of values between 12 and 17%. The saturation of hydrocarbons exhibits a range of values, spanning from 72 to 92%.

By employing several rock typing methodologies, such as the analysis of petrography data, RQI, FZI, R35, HFUs, and SML plots, seven distinct rock types were successfully discerned which characterized by their unique lithological compositions and flow properties. By employing porosity–permeability relationships, the equation formulated for each kind of rock can be utilized to predict the permeability at uncored intervals within the same well or even in other wells lacking core samples.

By taking into account the petrophysical characteristics of each flow unit, it becomes more straightforward to choose the most productive unit for perforation. It is crucial to incorporate equations that can forecast permeability values at regular intervals in the absence of core samples. This highlights the need of integrating well logs and core data.

## Data Availability

The data that supports the findings of this study are available from the corresponding author upon reasonable request.
